# The Impact of Fermented Scald on Rye and Hull-Less Barley Dough and Bread Structure Formation

**DOI:** 10.3390/foods12244475

**Published:** 2023-12-14

**Authors:** Ruta Murniece, Sanita Reidzane, Ruta Galoburda, Vitalijs Radenkovs, Dace Klava

**Affiliations:** 1 Faculty of Agriculture and Food Technology, Latvia University of Life Sciences and Technologies, Riga Street 22, LV-3004 Jelgava, Latvia; sanita.reidzane@lbtu.lv (S.R.); ruta.galoburda@lbtu.lv (R.G.); dace.klava@lbtu.lv (D.K.); 2 Processing and Biochemistry Department, Institute of Horticulture, Graudu Street 1, LV-3701 Dobele, Latvia; vitalijs.radenkovs@lbtu.lv; 3 Division of Smart Technologies, Research Laboratory of Biotechnology, Latvia University of Life Sciences and Technologies, Riga Street 22b, LV-3004 Jelgava, Latvia

**Keywords:** rye, hull-less barley, fermentation, scald, gelatinized starch, microstructure, rheology, texture

## Abstract

In wholemeal bread production, scalding and fermentation contribute to the improvement of the structural characteristics of the dough and bread. The influence of fermented scald on rye and barley dough and bread structure formation was specified in this study. The microstructural analysis performed using a scanning electron microscope revealed the separation of phases during the fermentation of scalds. According to the storage G′ and loss G″ moduli, both scalds exhibited elastic character over viscous. The fermentation of barley scald increased both moduli and complex viscosity, while no substantial changes were observed in the fermented rye scald. The addition of fermented scald containing partially hydrolyzed starch and a fraction of water-soluble compounds contributed positively to the formation of a well-organized structure of dough fermented for 4 h. Fermentation substantially reduced the dough’s complex viscosity and moduli values, confirming the partial structure alteration leading to the viscous portion increase. The dough with fermented scald showed a significantly lower loss factor than the dough without fermented scald, indicating enhanced mechanical process ability. The most substantial weakening of the structure was observed for dough without scald. The addition of rye scald to the rye dough promoted the formation of fewer pores with relatively smaller specific volumes.

## 1. Introduction

The wholemeal rye and barley dough structure represents a complex multicomponent system of starch and non-starch polysaccharides, proteins, and lipids. The presence of an extensive amount of dietary fiber adversely affects the structure-forming process during rye and barley bread-making. In addition, arabinoxylans in rye and *β*-glucans in barley demonstrate high water-binding capacity and the ability to form viscous solutions [1,2]. Considering these aspects, to a certain extent, wholemeal flour bread production presents a challenge. On the other hand, wholemeal polysaccharides possess technological functionality as they contribute to the overall rheology and exhibit gelling properties with texturizing potential [3,4,5].

Subjecting the starch granules to thermal processing in the presence of water results in the swelling and leaching of macromolecules into surrounding fluids, affecting the viscosity and gel formation [6]. The water-soluble non-starch polysaccharides such as arabinoxylans, in turn, entrap proteins, lipids, and starch granules, thus contributing to the strengthening of the air–water interfacial forces of certain dough liqueurs, such as those made of rye [7]. The positive effect of non-starch water-soluble polysaccharides due to their strong hydration ability was reported to substantially affect flour’s water absorption, dough yield, bread volume, moisture content, and hardness of the bread crumb [5].

Using the scalding technique in preparing wholemeal bread contributes to achieving sufficient structural characteristics of the dough and bread crumb. It benefits the bread-making process, positively contributing to delaying the staling rate. Making wholemeal rye bread with scalded rye flour is typical in Europe’s northern and eastern regions, including the Baltic countries [8]. The application of scalded rye flour in bread-making is reported to have a beneficial effect on the plasticity of both the crumb and the crust, along with bringing a pleasantly sweet-and-sour taste to the bread. It has also been observed that the formation of dough liquor rich in mono- and disaccharides, such as glucose, fructose, and maltose, during flour scalding, as well as partially hydrolyzed starch granules, delays staling and improves sensory properties and overall acceptance of the wheat-rye bread [9]. As the flour processing technique, scalding attracts researchers worldwide, including Japanese and Chinese, as it positively contributes to forming functional and health-promoting components [10,11].

Scalding refers to the technological process of pouring hot water (90–98 °C) over the flour to prepare sourdough bread [12]. This process makes starch more accessible to *α*- and *β*-amylases by breaking down intermolecular bonds between starch molecules, called starch gelatinization. The effectiveness of scalding in inhibiting potentially harmful microbiota, suppressing the activity of lipolytic enzymes involved in the conversion of lipids into odorous substances, and promoting starch accessibility to enzymatic attacks has been reported [13]. The crumb softness was reported to be improved by including gelatinized and well-enough-cooled starch [14]. Saccharification and liquefaction of the starch occur within 2–4 h at 62–65 °C right after starch gelatinization. The processes of saccharification and liquefaction ensure the availability of simple sugars from the complex starch molecules, which is due to the action of endogenous amylolytic enzymes [15]. The hydrolysis of gelatinized starch causes a marked increase in the content of fermentable saccharides in the dough. In a moderate amount, these sugars are considered a superb substrate for developing lactic acid bacteria (LAB) during further fermentation [9,16]. Moreover, the formed sugars were reported to contribute significantly to the dough rising triggered by yeasts [17]. It has been reported that thermophilic LAB is suitable for thermophilic sourdough production [18]. *Lactobacillus delbrueckii* belongs to the group of obligately homofermentative lactobacilli, and they produce lactic acid as the sole main endpoint of carbohydrate metabolism [19]. The presence of *Lactobacillus delbrueckii* as a dominant microbiota in spontaneous rye sourdoughs has been documented by Kulp [20] and Kozlinskis [21]. Since the ratio of sweet to sour taste in wholemeal bread and the distinguished flavor are key factors affecting consumers’ acceptance, the proper selection of LAB is crucial in achieving the desired sensory quality of the final product [9].

In preparing wholemeal rye bread, the lack of proteins responsible for forming a protein network similar to wheat gluten makes the bread-making process challenging. Like rye, the use of hull-less barley flour in bread making due to its lack of gluten, poor baking properties, and low sensory quality makes barley grains less attractive for bakery products [22]. Moreover, it has been reported that high-molecular-weight β-glucans decrease the water availability for the gluten network and thus impair the baking properties [23]. According to Bhatty’s [24] observation, adding as much as only 10% of barley flour to the recipe did not affect the quality of leavened bread, whereas increasing the ratio of *β*-glucans to flour adversely affected the physical properties of bread. However, a two-step process, that is, flour scalding and fermentation, can lead to achieving the desired structural properties and sensory quality of the bread crumb [9,25]. Previous research dealing with scalded flour has already confirmed the positive effect of scalding on the accumulation of fermentable sugars, reduced acrylamide content, and extended shelf-life of rye and barley breads. A positive impact of scalded rye flour on the physical and structural properties of rye bread was reported by Esteller and Lannes [26], highlighting that adding scalded flour promoted homogenous, interconnected pore formation with relatively smaller cell area, diameter, and perimeter, thus ensuring heat and fluid transport during the bread-baking process.

The scientific evidence available to date is mainly related to assessing the impact of scalded flour on the physical properties of bread; however, the textural characteristics of scalded flour and sourdough bread are generally underestimated. The lack of scientific literature promoted the undertaking of this study, focusing on the characterization of the microstructural and rheological properties of rye and barley scalded flour and their effect on rye and barley bread’s physical properties.

## 2. Materials and Methods

### 2.1. Materials

#### 2.1.1. Rye and Barley Flour and Rye Malt Characteristics

The commercial rye (*Secale cereale* L.) flour of the Kaupo grain variety and hull-less barley (*Hordeum vulgare* L.) flour (further in text, barley flour) of the Kornelija grain variety were obtained from the farm Kanepites (Latvia). Both cereal grain types were harvested in 2022. The flour’s proximal composition is depicted in Table 1 (obtained from the producer). A high content of β-glucan (5.57% dw) in Kornelija hull-less barley flour was also observed in our previous study [27].

Rye flour (moisture 14.69 ± 0.30%) and barley flour (moisture 13.79 ± 0.30%) were passed through a series of sieves with a mesh diameter ranging from 160 µm to 450 µm using a vibratory sieve shaker AS 200 (Retsch GmbH, Haan, Germany). The distribution of particles in rye flour was found as follows: above 450 µm—11.32%, 315 µm—22.25%, 250 µm—17.89%, and 160 µm—18.61%. The distribution of particles in barley flour was found as follows: above 450 µm—2.15%, 315 µm—27.17%, 250 µm—18.44%, and 160 µm—24.37%.

Rye diastatic malt with a diastatic power (DP) of 267 WK and a moisture content of 4.52% was purchased from Latmalt Ltd. (Jelgava, Latvia).

#### 2.1.2. Sourdough of Rye and Barley Scald

A 24 h fermented part of fermented scald (Sourdough S) received from the bakery Kelmeni (Ranka, Latvia) was used for scald fermentation. The prevalence of *Lactobacillus delbrueckii,* as the most widespread lactic acid bacteria (LAB) representative, was identified in fermented rye and barley scald using polymerase chain reaction-amplified variable regions of 16S rRNA.

Reviving and maintaining Sourdough S was performed daily in the bakery by exchanging part of the fermented scald with a new portion of the scald. Scald was prepared as follows: water and flour were mixed with hot water (96 °C) and slowly cooled down to 55–60 °C for 2 h, and after inoculation with a leftover portion of fermented scald, daily renewal was performed.

### 2.2. Fermented Scald Preparation

Experiments were carried out in the laboratories of the Food Institute, Latvia University of Life Sciences and Technologies. Fermented scald was prepared according to the technology used in the industry/bakery, slightly modified, and adapted to the conditions in the laboratory environment.

Rye flour scald (RS) and barley flour scald (BS) were prepared separately (DY-350, DY = (flour weight + water weight)100/flour weight). To prepare scald, 1400 g of flour and 14 g (1% of the flour mass) of diastatic rye malt were mixed with 3500 g of hot water (96 °C). The prepared scald was well-mixed for 10 min using a mixer, Teddy (A/S Varimixer, Brøndby, Denmark). The saccharification process was ensured by keeping the mixture in the climate chamber Memmert ICH 110 (Memmert GmbH, Schwabach, Germany) for 90 min at 60 °C and at a relative humidity of 80%, thus ensuring slow scald cooling from 75 to 60 °C. Afterwards, 84 g (6% of the flour weight) of Sourdough S provided by the bakery Kelmeni was added to the scald. Fermentation was performed in a climate chamber at a relative humidity of 60%, ensuring a gradual temperate decrease for the continuous dough cooling using the following modes: at 55 °C for 5 h, at 50 °C for 5 h, at 45 °C for 5 h, and at 35 °C for 5 h. Finally, the dough was maintained at room temperature (22 ± 2 °C) to reach 30 °C (approx. 2 h). Rye and barley scald (RS and BS) and fermented scald (FRS and FBS) samples were analyzed, and fermented scald was used for dough preparation immediately after fermentation. Individual scald and fermented scald samples were frozen and lyophilized for further microstructure analysis.

### 2.3. Rye Sourdough Preparation

Rye sourdough (Sourdough D) was prepared and used further to ferment the dough. Sourdough D (DY-150) was prepared under laboratory-controlled conditions. LAB pure cultures, that is, *Lactiplantibacillus plantarum* DSMZ 20205, *Pediococcus pentosaceus* LMKK 773, and *Lactococcus lactis* subsp. *Lactis,* and yeast *Naumovozyma dairensis* LMKK 575, were obtained from the Microbial Strain Collection of Latvia (MSCL, University of Latvia, Riga, Latvia).

Pure cultures of microorganisms were obtained in De Man, Rogosa, and Sharpe (MRS) selective culture media on Petri plates in anaerobic packaging and were stored at 4 ± 1 °C until use. Yeasts were obtained in malt extract agar (MEA) on Petri plates. The test cultures were subcultured into freshly prepared MRS (Biolife, Milan, Italy) for activation and incubated for 24 h at 37 °C. Yeasts were activated in malt extract agar (MEA) (Biolife, Milan, Italy) for 24 h at 25 °C. After incubation, cultured microorganisms were transferred to a 10 mL saline solution until the number of LAB and yeast colony-forming units (CFU) reached 10^9^ and 10^7^ CFU in 1 mL, respectively. Further, to a 10 mL saline solution containing cultured LAB, 30 g of rye flour and 60 g of rye scald were added, followed by the incubation of the mixture for 5 h at 31 ± 1 °C. Then, 10 mL of suspension containing yeast was added to the prepared mixture, followed by 40 g of rye flour and 90 g of rye scald. The mixture was further fermented for 14 h at 28 ± 1 °C. Finally, 180 g of rye flour and 90 g of ultra-pure water were added to the prepared mixture and fermented for an additional 12 h. The last step was repeated twice.

### 2.4. Dough Preparation and Bread Making

Four types of dough were prepared: barley dough with fermented scald (BDWS), barley dough without fermented scald (BDNS), rye dough with fermented scald (RDWS), and rye dough without fermented scald (RDNS). The ingredients of the dough are specified in Table 2. The formula was modeled to maintain an equal flour-to-water ratio in the dough prepared with and without the scald. Dough kneading for 15 min at 300 rpm (speed 2) was performed using a planetary dough mixer, the Varimixer Bear AR 10 (A/S Wodschow & Co. Brøndby, Denmark).

After dough preparation, it was fermented in the climate chamber Memmert ICH110 (Memmert GmbH, Schwabach, Germany) for 2 h at 32 ± 1 °C and a relative humidity of 80%. Afterwards, the obtained dough was divided into equal pieces of 400 g each, placed in forms, and fermented for an additional 2 h in the climate chamber. Samples for dough analysis were taken and analyzed immediately. Individual dough samples were frozen and lyophilized for further microstructure analysis using a scanning electron microscope (SEM). The bread was baked for 30 min at 200 ± 2 °C in an S400 Mini Rack oven (Sveba Dahlen AB, Fristad, Sweden). An internal crumb temperature of 98 °C was reached for all samples. The bread was stored in the laboratory room and covered with 12.7 µm thick cling wrap. Individual bread samples were frozen and lyophilized for further microstructure analysis by SEM.

**Figure 1:**
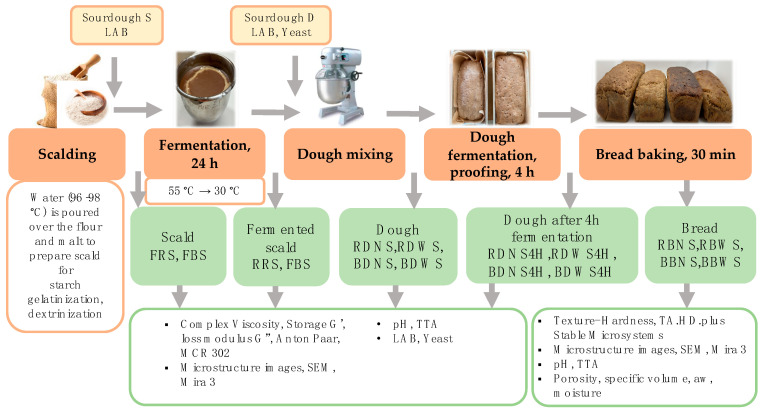
A schematic representation of major steps undertaken to prepare scald, fermented scald, dough, fermented dough, and bread, along with their further analysis.

The scald, dough, and bread were lyophilized in a vacuum freeze-dryer FT33 MkII (Armfield Ltd., Hampshire, UK) for 72 h, maintaining 6.4 ± 0.1 Pa and −40 °C in a condenser chamber. A schematic representation of the study design is depicted in Figure 1.

### 2.5. Lactic Acid Bacteria and Yeasts Enumeration of Rye and Barley Scald and Dough

The enumeration of microorganisms was performed by the plate count method [28,29], whose cultivation was implemented using relevant media and broth. Media preparation was accomplished according to the ISO 11133-1:2009 standard [30]. Sample dilutions were prepared using a saline solution following the protocol described in ISO 6887-1:2017 [31]. For the cultivation of LAB, MRS medium (Biolife, Milan, Italy) was used. The LAB was incubated in a jar under anaerobic conditions for 72 h at 37 ± 1 °C using an oxygen absorbent BD GasPak EZ (Benex Ltd., Dublin, Ireland). For the cultivation of yeasts, MEA (Biolife, Milan, Italy) was used. The Petri plates were incubated for 48 h at 25 ± 1 °C. Microorganisms were incubated in the Memmert IPP200 incubator (Memmert, GmbH, Schwabach, Germany).

The total microorganism colonies were estimated using the automatic colony counter Scan 500 (Interscience, Bois des Arpents, France) and represented as colony-forming units (CFU) log_10_ CFU g^−1^.

### 2.6. Microstructure Study Using SEM

After fermentation with LAB, the microstructure of lyophilized rye and barley scalds and the dough was analyzed using a Mira3 scanning electron microscope (SEM) by Tescan Orsay Holding, a.s. (Brno, Czech Republic). The test specimens were mounted onto SEM pin stubs using double-sided adhesive carbon discs, and non-fixed material was blown by a gentle stream of N_2_ and coated with a gold-palladium (80/20, respectively) 15 nm thick layer using a high vacuum spotter coater, the Leica EM ACE600 (Leica Microsystems, Wien, Austria). The conditions were adjusted to operate in high-vacuum mode using the secondary electron (SE) and back-scattered electron (BSE) detectors. The morphology of samples was analyzed by increasing magnification up to 2500× for precise dimension measurement while operating at a 5 kV acceleration voltage.

The microstructure of bread was analyzed using the digital microscope 3D Hirox RH-2000 (Hirox Europe, Limonest, France) equipped with a high-range triple zoom lens MXB-5000REZ under 140× magnification.

### 2.7. Assessment of Rye and Barley Scald and Dough Rheological Properties

The rheological properties were analyzed using the MCR 302 rheometer (Anton Paar, Graz, Austria) with a 25 mm diameter parallel plate measuring system (gap 1 mm). An angular frequency sweep from 100 to 1 rad s^−1^ was performed at a 0.1% strain and detected within the linear viscoelastic region. The strain value for frequency sweep was chosen within the previously determined viscoelastic region (LVR). This region was identified through the amplitude sweep test, conducted at a frequency of 10 Hz, where the strain was increased from 0.01% to 100%. The temperature was kept at 30 °C using the Peltier module. Complex viscosity (Pa s), storage G′ and loss G″ moduli (Pa), and loss factor were analyzed in duplicate from the two independent batches.

Analysis of scald and dough texture was performed using a TA.HD.Plus texture analyzer (Stable Microsystems, Godalming, UK). The scald and dough were evaluated using the A/BE-d40 back extrusion rig (40 mm in diameter), moving at 1 mm s^−1^ to a penetration depth of 15 mm. The samples were filled in the cup up to 30 mm in height. A trigger force was set at 0.049 N and a temperature of 20 ± 1 °C. Every batch was analyzed in two replicates. The original software Texture Exponent 32 (Stable Microsystems, Godalming, UK) measured the peak positive force (N) required to penetrate scald and dough.

### 2.8. Determination of Chemical and Physical Parameters of Rye and Barley Dough and Bread

The moisture content was determined gravimetrically using the Precisa XM 120 moisture analyzer (Precisa Gravimetrics AG, Dietikon, Switzerland). The water activity is estimated by the water activity meter Labmaster-aw Neo (Novasina AG, Lachen, Switzerland). The pH of sourdough was determined according to the AACC 02-52:1999 [32] method using a pH meter (Mettler Toledo, Germany).

Total titratable acidity (TTA) was measured according to the AACC 02-31.01 method [33].

The bread volume was determined by rapeseed displacement according to the AACC Method 10-05.01 [34]. Specific volume was calculated as a loaf volume and bread weight ratio. Bread porosity was determined using the Zhuravlev device (Biomer Ltd., Krasnoobsk, Russia) according to a method previously described by Cizeikiene [18].

Analysis of bread texture was performed using a TA.HD. Plus texture analyzer (Stable Microsystems, Godalming, UK), according to AACC 74-09 [35], with some modifications. The trigger force was set at 0.049 N. Two slices of mechanically sliced bread (each 7 mm thick) were compressed with a 25 mm aluminum cylindrical probe at 1.7 mm s^−1^ speed. Peak positive force (N) describes bread crumb hardness, and peak negative force (N) describes stickiness. Analysis was performed 24 h after baking. The results are the means of ten replicates.

### 2.9. Statistical Analyses

The levels of the significant effects were mutually compared using Tukey’s Honest Significant Differences post-hoc test. A visual assessment of diagnostic plots ensured that the data conformed to the statistical assumptions. The statistical analysis was conducted in R v. 4.2.3 [36].

## 3. Results

### 3.1. Microbial and Physico-Chemical Characteristics of Scald and Sourdough

The estimation of LAB and yeasts, as well as the physico-chemical characteristics of fermented scald, revealed that after 24 h of fermentation, there was a significant (*p* ≤ 0.05) increase in the number of LAB and yeasts (Table 3). As seen, the increase in the amount of LAB in fermented rye scald (FRS) and fermented barley scald (FBS) after 24 h of fermentation corresponded to 7.4 and 6.0 log_10_ CFU g^−1^, respectively. The difference in LAB between fermented and non-fermented rye and barley scald was 2.5 and 1.5 log_10_ CFU g^−1^, respectively, indicating that both scalds are preferable substrates that ensure the necessary composition of carbon-containing compounds for LAB viability. Fermentation of scald by LAB *L*. *delbrueckii* resulted in the desired taste and aroma specific to fermented scald bread, along with the formation of lactic acid [37]. The increase in LAB count has led to a decrease in the pH of fermented rye and barley scalds. The decline in pH and rise in titratable acidity (TTA) are conditioned by the increase in metabolites, mainly lactic acid, produced by LAB.

As a result of flour scalding, native microorganisms, including bacteria, yeast, and mold present in flour, are getting inactivated [13]. Sourdough S was added to the scalded flour to ensure proper fermentation. The results indicated that both scalds contained a sufficient amount of LAB, typical for sourdough. A relatively high temperature for both scalding and fermentation negatively affected the growth and viability of yeast. The number of yeasts in both scalds largely remained unaffected by fermentation time. The number of yeasts in RBS and FBS was insufficient to be used as a leavening agent in bread preparation. In this regard, the Sourdough D with a high yeast CFU was used for the leavening function for further experiments.

### 3.2. Microstructure of Rye and Barley Scalds by Scanning Electron Microscope

**Figure 2:**
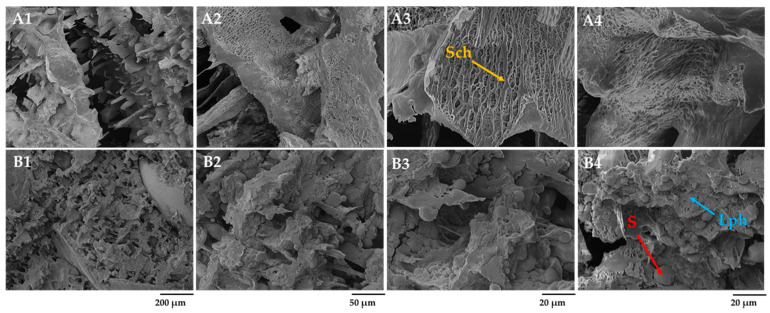
Effects of gelatinization and saccharification on the microstructure of scalded rye flour: (**A1**–**A4**) scalded rye flour after 2 h of gelatinization and saccharification (RS), (**B1**–**B4**) rye scald after 24 h of fermentation with Sourdough S (FRS), (**A3**)—starch amylose and other soluble components, and (**B4**)—phase separation-amorphous starch granules (S) in the liquid phase (Lph) of amylose, soluble arabinoxylans, proteins, and other soluble components. Sch—saccharified viscous liquid phase magnification 250× (200 µm), 1.0 k× (50 µm), and 2.5 k× (20 µm).

Figure 2 illustrates the formation of structure and changes in rye and barley flour scalds after 2 h of gelatinization and saccharification (RS and BS) and after 24 h of fermentation with starter (FRS and FBS). A gelatinized, viscous mass termed a starch paste was formed in the scalded and saccharified flour (Figure 2A4). When heated above the crystallization temperature, the crystallites inside the starch granules begin to melt, amylose leaches [38], and irreversibly swells when absorbing water. Mainly, starch amylose dissolves in water, forming an amylose matrix. Upon cooling, the starch gel is formed by dispersing granules in water. No visible signs of starch grains were observed on the surface of the rye scald after 2 h of gelatinization. The added malt with α-amylase activity facilitated the breakdown of starch into fermentable sugars. Klupsaite et al. [9] have confirmed the fructose and glucose formation during rye wholemeal flour’s scalding, while non-scalded wholemeal flour lacks such sugars. Klupsaite et al. also highlighted that the content of maltose had increased almost six times in scalded flour compared to the non-scalded ones. The uniform distribution of the saccharified (Sch) glucose, fructose, and resistant dextrin networks over the entire surface can be seen on micrographs. After 24 h of scalded rye flour fermentation with LAB-containing starter (FRS), the lenticular starch granules, which were not hydrolyzed, were swollen and appeared more pronounced on the surface (Figure 2B1–B4). Two fractions of overripe starch granules with an average size ranging from 5 to 20 µm entrapped in the solid phase of amylose and other soluble substances can be observed. A similar honeycomb-like network structure of scalded wheat flour has been reported by Li et al. [13], indicating that the leaching of starch chains during thermal exposure and air wrapping into the dough during dough mixing were the primary causes.

After 24 h, the matrix of scalded rye flour represented no uniform order. The non-hydrolyzed starch granules were integrated into a viscous substance, forming an amorphous network (Figure 2B2,B3). It has been reported that on cooling, the amylose gels form an amylose network surrounding the swollen starch granules [4]. The granules act as fillers in the amylose network, forming a phase-separated gel. This statement can be reinforced by observations in the current study revealing structure-forming phase separation that can be seen in the SEM micrograph (Figure 2B4). As indicated, non-hydrolyzed starch represents a solid phase, while gelatinized starch, water-soluble dietary fiber, such as arabinoxylans and *β*-glucans; fructans; and denatured protein represent a liquid phase (Lph).

**Figure 3:**
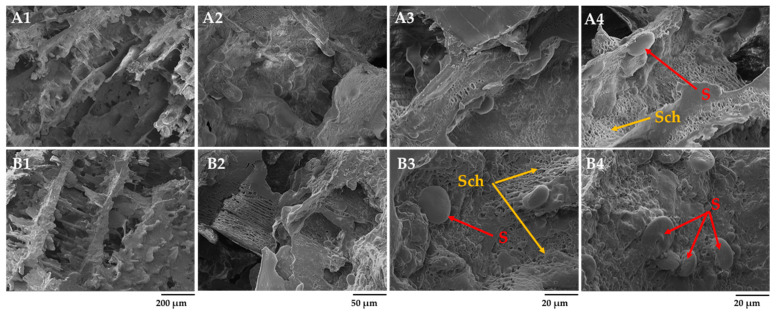
Effects of gelatinization and saccharification on the microstructure of barley scald: (**A1**–**A4**) scalded barley flour after 2 h of gelatinization and saccharification (BS) and (**B1**–**B4**) barley scald after 24 h of fermentation with Sourdough S (FBS). Sch—saccharified viscous liquid phase; S—swollen lenticular spherical starch granules; magnification 250× (200 µm), 1.0 k× (50 µm), and 2.5 k× (20 µm).

The microstructure analysis of scalded barley flour after 2 h gelatinization and saccharification revealed the presence of starch granules (S) in saccharified or dextrinized form covered with the liquid phase of fermentable sugars and other water-soluble components (Sch) (Figure 3B4). After 24 h of scalded barley flour fermentation with LAB-containing Sourdough S, the swollen lenticular starch granules became more apparent. The liquid phase of scalded barley flour differed from that of rye scald, mainly by the size of starch granules and the more heterogeneous surface. Like rye scald, barley scald was represented by two fractions of starch granules, though the size of the granules varied from 1 to 30 µm. Since amylose rather than amylopectin takes precedence in the starch of barley grain, making it more resistant to α-amylase attacks (resistant starch), the relative volume of starch granules in scalded barley flour was found to be substantially higher than that observed in scalded rye flour [39].

### 3.3. Rheological Properties of Rye and Barley Scald

**Figure 4:**
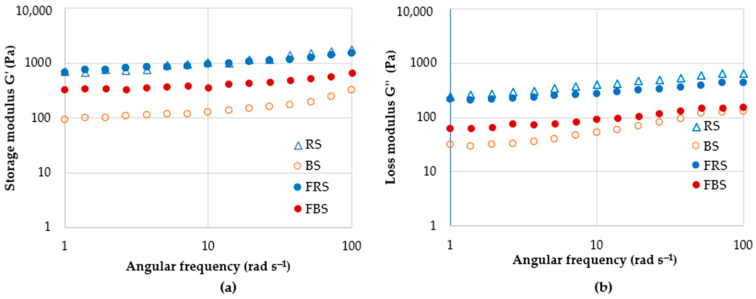
Storage G′ (**a**) and loss G” (**b**) moduli of rye (RS) and barley (BS) scald and fermented rye (FRS) and fermented barley (FBS) scald.

In all scalds studied, the storage modulus (G′), representing the elastic character, was higher than the loss modulus (G″), representing the viscous character (Figure 4). Other researchers have also observed the prevalence of elastic properties over viscous in gelatinized barley starch [40]. The effect of fermentation significantly differed in rye and barley scald. The rheological properties of gelatinized starch depend on various factors, such as the chemical structure of starch, gelatinization temperature, and time. In rye scald, fermentation resulted only in a slightly increased G″ value, whereas G′ did not change. Contrarily, both storage and loss moduli increased in barley scald upon fermentation; this may be due to the swelling of starch grains and the formation of water-soluble fractions leaking into the liquid phase. It might be promoted by the increased temperature applied in the scalding process. Punia [40] observed an improved barley starch swelling ability at 50–60 °C and increased solubility at 80–90 °C. Thus, during fermentation, the scald temperature gradually decreased from around 55 °C to 30 °C, which was favorable for further starch swelling. As a result of heating, the starch swells, and the crystalline structure is disrupted, contributing to the binding of water molecules and increased solubility. Enzymatic degradation of starch and dextrin formation initiated by scalding is stopped when pH decreases in the fermentation.

**Figure 5:**
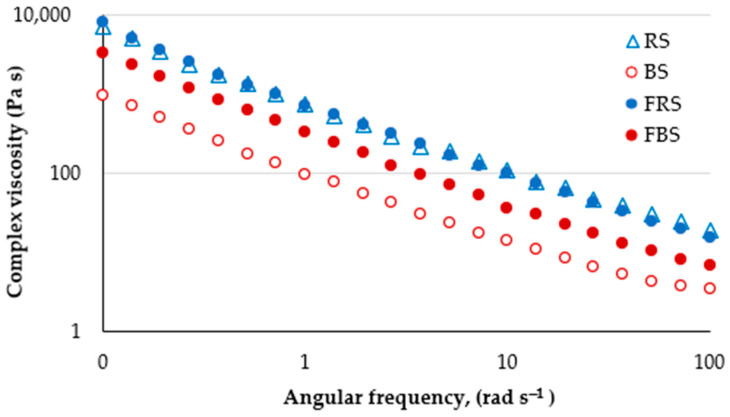
Complex viscosity of the rye scald (RS), barley scald (BS), fermented rye (FRS), and fermented barley scald (FBS).

The complex viscosity of rye scald was higher than that of barley scald (Figure 5). For all scalds, the viscosity decreased with increased oscillation frequency, possibly due to shear-induced structural changes. The 24 h fermentation did not affect the rye scald’s viscosity, whereas the barley scald’s complex viscosity was significantly increased. The high viscosity of barley scald may be contributed by β-glucans, which form slimy mucous substances in water solutions. Along with the starch swelling, an increase in complex viscosity and viscoelasticity values were observed in the FBS. At an elevated temperature of 38 °C, some 38–69% of β-glucans dissolve rapidly within 2 h [41], ensuring increased viscosity.

Several factors determine the viscosity of the gelatinized paste. Thus, the activity of the enzyme α-amylase leads to starch breakdown. The stability of gas pore membranes is determined by gelatinized starch and partially hydrolyzed starch, pentosans, proteins, and starch granules. If these membranes do not form rigidly and the scald is over-liquefied or starch is too degraded, then there is a possibility that the gas will escape and the structure will not be formed. The swelling characteristics of starch grains are primarily determined by the viscoelastic behavior of gelatinized dispersions [42]. Swollen starch granules are partially soluble due to amylose. Water-soluble arabinoxylans-pentosans have a higher water absorption capacity than water-soluble proteins and form viscous solutions.

The complex viscosity of fermented scald affects its water absorption capacity, which in turn affects the rate and extent of fermentation. Optimal fermentation dynamics are achieved when scald is fermented with consistent fluidity. This steady process ensures the uniform generation of acidity and aroma, creating a favorable environment for the proliferation of LAB. Under practical production conditions, this evenly fermented scald exhibits improved compatibility with mechanical processing.

### 3.4. Microstructure of Barley and Rye Dough with and without Fermented Scald before and after 4 h Fermentation

**Figure 6:**
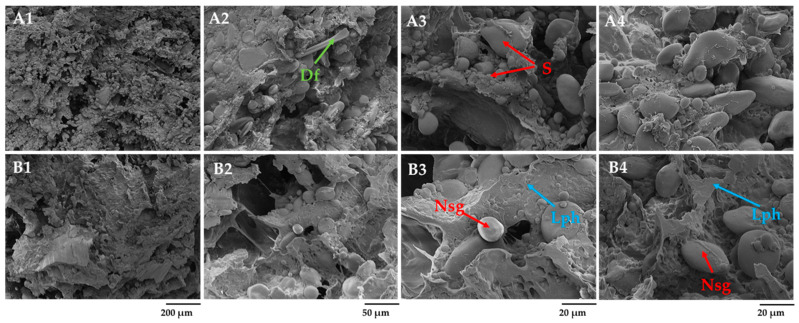
Effect of fermented scald incorporation on the microstructure of rye dough after kneading: (**A1**–**A4**) rye dough (RDNS) and (**B1**–**B4**) rye dough with fermented rye scald (RDWS). DF—fibrils of dietary fiber; Nsg—naked starch granules; Lph—liquid phase; S—swollen lenticular spherical starch granules; magnification 250× (200 µm), 1.0 k× (50 µm), and 2.5 k× (20 µm).

The scalded flour’s amylose matrix, with dissolved arabinoxylans and denatured proteins, created a ready-made structural network in the dough with scalded rye flour (Figure 6B1–B4). The addition of scalded rye flour to the dough seems to improve the kneading efficiency, as a more homogenous structure of the dough with an almost complete covering of the starch granules was observed. It has been documented that scalded material in the dough can ensure plasticity for both the crumb and the crust, making the kneading of the dough more convenient [13]. Swollen starch granules of different sizes and unformed liquid phases were observed in the dough without scald (Figure 6A1–A4).

**Figure 7:**
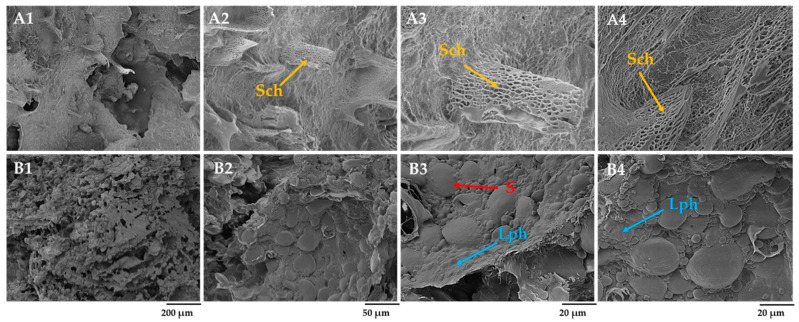
Microstructure of rye dough after fermentation for 4 h: (**A1**–**A4**) rye dough after 4 h of fermentation with Sourdough D (RDNS4H) and (**B1**–**B4**) rye dough with fermented rye scald after 4 h of fermentation with Sourdough D (RDWS4H). Sch—saccharified viscous liquid phase; Lph—liquid phase; S—swollen lenticular spherical starch granules; magnification 250× (200 µm), 1.0 k× (50 µm), and 2.5 k× (20 µm).

After 4 h of fermentation, the structure of the rye dough without scalded rye flour can be characterized as homogenous with distinctive and uniform network distribution over the entire surface (Figure 7A2–A4). It is speculated that the degradation of starch caused by the activity of LAB and yeast present in sourdough promoted the formation of networks composed of solubilized dextrin, maltose, and, to a lesser extent, glucose and other minor saccharides. The lack of naked and even covered starch granules in the dough matrix supports this observation. The ability of *L*. *plantarum* to hydrolyze barley starch granules within 48 h was reported by Xie et al. [43], indicating a relatively high volume of damaged starch grains with eroded surfaces. The availability of damaged starch granules gave rise to maltose, the primary carbon source for *S*. *cerevisiae* [44].

On the other hand, the dough with scalded rye flour contained swollen starch granules (S), gelatinized starch, and liquid phase (Lph) (Figure 7B3). The addition of scalded flour formed a solid, paste-like matrix, thus preventing its further depletion. Moreover, no apparent signs of gluten fibrils were observed in the dough after fermentation (Figure 7B1–B4), indicating different mechanical and physical properties of rye and barley proteins than those for wheat proteins. Rye proteins are mainly represented by two fractions, that is, albumin and globulin, which are soluble in water and reported to be more readily hydrolyzable under sourdough fermentation than the glutenin fraction of wheat protein [44].

**Figure 8:**
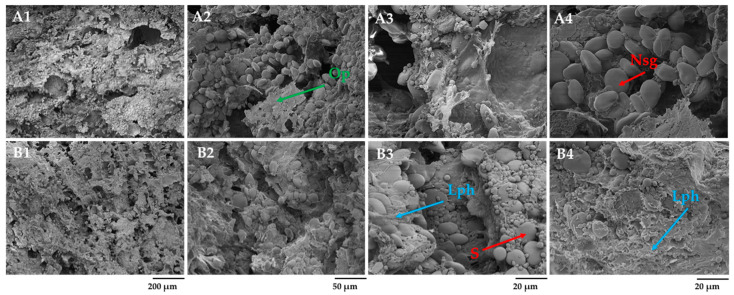
Effect of fermented barley scald incorporation on the microstructure of barley dough after kneading: (**A1**–**A4**) barley dough (BDNS) and (**B1**–**B4**) barley dough with fermented barley scald (BDWS). Lph—liquid phase; Nsg—naked starch granules; Op—grain’s outer parts; S—swollen lenticular spherical starch granules; magnification 250× (200 µm), 1.0 k× (50 µm), and 2.5 k× (20 µm).

After kneading, barley dough without scalded barley flour contained observed elongated and, to a greater extent, naked (Nsg) and tightly attached starch granules and strands of fibers (Figure 8A1–A4). The presence of the barley grain’s outer parts (Op) caused the formation of elongated and non-uniform pores, as reported by Albasir [45]. However, the addition of scalded barley flour to the dough promoted the formation of a well-organized honeycomb-like network with tightly attached starch granules embedded into the liquid phase (Lph) composed of amylose, soluble arabinoxylans, proteins, and other soluble components of scalded barley flour. Like rye-scalded-fermented dough, barley dough demonstrated no gluten network.

**Figure 9:**
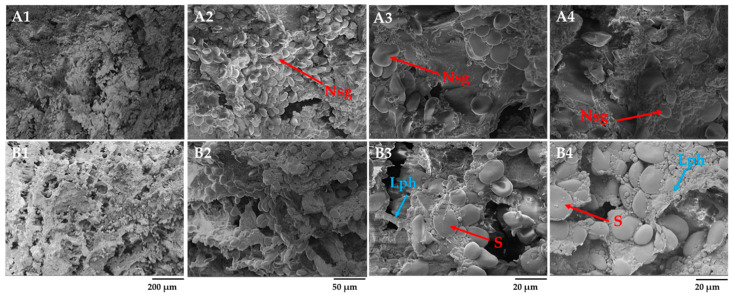
Effect of fermented barley scald incorporation on the microstructure of barley dough after fermentation for 4 h: (**A1**–**A4**) barley dough after 4 h of fermentation with Sourdough D (BDNS4H) and (**B1**–**B4**) barley dough with fermented barley scald after 4 h of fermentation with Sourdough D (BDWS4H). Nsg—naked starch granules; Lph—liquid phase; S—swollen spherical starch granules; magnification 250× (200 µm), 1.0 k× (50 µm), and 2.5 k× (20 µm).

After 4 h of fermentation, the structure of the barley dough without and with scalded barley flour was found to be substantially different (Figure 9A2–A4,B1–B4). It was observed that the dough matrix fermented for 4 h generally presented an uneven mixture of spherical-shaped starch granules only partially integrated into the dough matrix. As expected, the addition of scalded barley flour promoted the formation of a well-organized dough matrix composed of tightly attached starch granules covered with liquid phase (Figure 9B2–B4) (Lph). After 4 h of dough fermentation, small and large fractions of starch predominated, possibly indicating the availability of readily fermentable constituents, such as maltose and glucose, suitable as a carbon source for yeast development.

Overall, the scalding of rye and barley flour based on SEM analysis positively affected the microstructure of the dough formulated. The addition of fermented scald containing partially hydrolyzed starch and fractions rich in water-soluble compounds such as dextrins, maltose, and denatured proteins made it attainable to get a well-organized structure of dough fermented for 4 h. The availability of fermentable sugars in the scalded flour likely ensured the carbon-containing nutrients for LAB and yeasts. The fermented scald preserved part of the starch fraction involved in forming a solid carcass of dough. No visible signs of the gluten network, neither in rye nor barley dough, were observed, indicating an utterly different interconnection of grain components to make a solid matrix.

### 3.5. Rheological Properties of Rye and Barley Dough without Fermented Scald and with Fermented Scald

**Figure 10:**
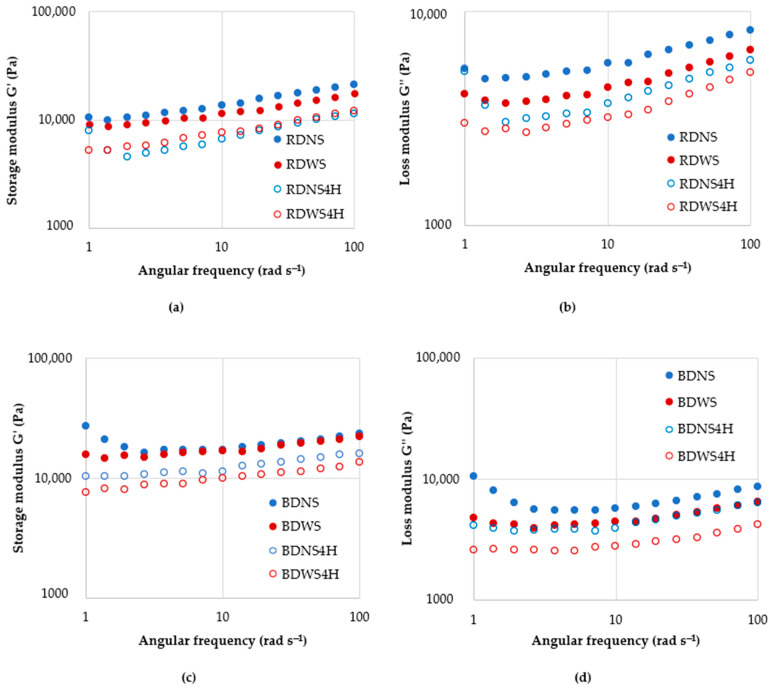
Storage G′ ((**a**)—rye dough, (**c**)—barley dough) and loss G″ moduli ((**b**)—rye dough, (**d**)—barley dough) of rye (RDNS) and barley (BDNS) dough without fermented scald and rye (RDWS) and barley (BDWS) dough with fermented scald after mixing and after 4 h fermentation.

The storage G′ and loss G″ moduli curves show elastic and viscous properties (Figure 10). G′ for all doughs was higher than G″, indicating that elastic properties dominated over viscous properties. The prevalence of elastic properties over viscous properties was also observed for Taftan dough with barley flour and composite wheat flour dough [46]. The fermentation reduced both moduli. A similar effect of fermentation on doughs has been observed by other researchers [47].

The complex viscosity and moduli changes were analyzed for rye and barley doughs without added scalds (RDNS and BDNS) and with scalds (RDWS and BDWS). The highest viscosity was observed for barley dough, irrespective of frequency (Table 4).

Fermentation substantially reduced the dough’s complex viscosity and modulus values, confirming the partial structure alteration. RDNS showed a slightly lower viscosity after fermentation than RDWS, which indicates weaker intermolecular friction in weaker internal structures, leading to easier sliding. On the other hand, for barley dough, the lowest viscosity was observed for sample BDWS. The increase in loss factor observed upon dough fermentation implies that the viscous portion increased due to the partial degradation of starch and changes in the dough structure.

### 3.6. Physical and Chemical Characteristics of Wholegrain Rye and Barley Bread

**Figure 11:**
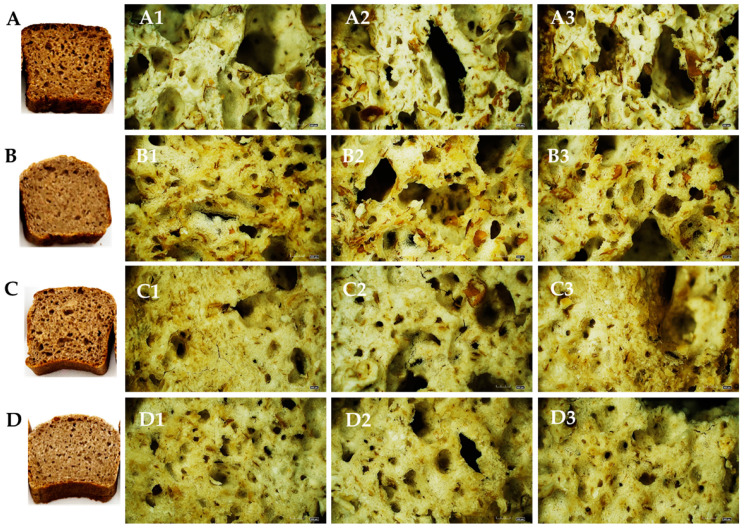
Cross-sections of the obtained bread crumbs: (**A**,**A1**–**A3**) rye bread without scald (RBNS), (**B**,**B1**–**B3**) rye bread with scald (RBWS); (**C**,**C1**–**C3**) barley bread without scald (BBNS), and (**D**,**D1**–**D3**) barley bread with scald (BBWS). The structure of bread samples was observed at 140 times magnification.

The physical and chemical characteristics, that is, pH, titratable acidity (TTA), water activity (aw), moisture, porosity, and specific volume of the bread made using rye and barley grain with and without scalded flour, are given in Table 5, as well as bread crumb images provided in Figure 11. The pH values of barley (BBNS) and rye (RBNS) bread without scald were significantly different (*p* ≤ 0.05); the pH values ranged from 4.89 to 5.15, respectively. However, the pH of rye and barley breads made with scald was significantly lower (*p* ≤ 0.05), corresponding to pH values of 4.09 and 4.26, respectively. The lower pH in bread with added scald indicates more effective dough fermentation due to the higher reducing sugar content, thus promoting more intense lactic acid production by LAB. Since the growth of LAB is accompanied by the production of lactic acid, along with a decrease in pH, the titratable acidity (TTA) of bread samples was also increased. The observed TTA in bread without scald ranged from 5.0 to 5.3 mL NaOH with no significant differences (*p* > 0.05), though the TTA in bread made with added scald was from 7.8 to 10.4 mL NaOH. The results indicated a relatively higher TTA value in bread prepared with rye flour and fermented rye scald (RBWS), which supports better lactic acid accumulation in this type of dough than in barley dough (BBWS).

The differences were observed between the water activity (a_w_) of bread without and with scald; the values ranged from 0.95 to 0.96. The observed a_w_ values of bread align with those reported by [48,49] for wheat and barley bread. However, significant differences (*p* ≤ 0.05) in the specific bread volume were observed between the bread samples prepared with and without fermented scald.

The results showed that adding rye scald promoted the formation of fewer pores with a relatively smaller specific volume (Table 5 and Figure 11). Adding a scald to the recipe influences the bread’s pore size, distribution, and shape. The formation of smaller pores can be explained by the stability of the structure and the less available starch granules that, during dough fermentation, are covered with the liquid phase of scald, thus restricting their water absorption and swelling.

Since pores in bread crumb act as a network array of capillary vessels separated by random empty spaces, their more even distribution over the crumb and interconnection would contribute to more effective heat and mass transfer during thermal exposure. All these changes in bread porosity positively affect water-holding capacity, liquid uptake, and bulk density. This statement can be partially supported by the findings of the current study since only the RBWS sample prepared with scald was found to be significantly (*p* ≤ 0.05) softer than that without scald (RBNS) (Table 5). A similar observation was made by Klupsaite [9], indicating the availability of arabinoxylans in rye flour, whose extractability and swelling capacity increase under acidic conditions, thus reducing the staling process through more effective water binding and forming a viscous dough structure. Contradictory results were observed in barley bread made with and without scald, where the hardness of the BBWS sample was 4.4 N higher than that of BBNS (Table 5). Reidzane et al. [27] have recognized the pronounced hardness of wholemeal barley bread in previous studies, and Cakir et al. [50] have also reinforced it. The structure of barley bread, with a smaller ratio of liquid phase and a more rigid arrangement of starch granules, as well as a higher viscosity, was reflected in the higher hardness of the bread. No direct relationships were observed between moisture content and the hardness of the rye and barley bread crumbs.

## 4. Conclusions

Substantial changes were observed in the structure of fermented scalded rye and barley flour and their impact on dough fermentation and bread crumb compared to wholegrain sourdough bread without scald. Rheological analysis revealed that rye- and barley-scalded flour behaved differently after saccharification and fermentation, mainly due to the flour’s composition and physico-chemical properties. An example is pH, which varied from 4.09 to 5.15 and from 4.26 to 4.89 for rye and barley bread with and without scald, respectively, indicating a more effective dough fermentation in the presence of scald with a high reducing sugar content. The study revealed distinct mechanisms of structure formation in doughs where a gluten network was not formed. Rheological investigations of rye and barley scald have been conducted, elucidating significant insights into their viscoelastic behavior. The effect of fermentation manifests differently between rye and barley scalds. For rye, marginal alterations in the loss modulus were recorded post-fermentation. In contrast, barley exhibited an elevation in the storage and loss moduli that can be attributed to the swelling of starch grains and the emergence of water-soluble fractions. In doughs subjected to sourdough fermentation, the primary role is assigned to the activity of native enzymes in the flour and the decomposition efficiency of complex compounds into simpler ones by microorganisms. Polysaccharide characteristics, such as retrogradation and saccharification, play pivotal roles in dough structure formation and serve as substrates for microorganisms’ development. Incorporating fermented rye scald in the bread dough positively affected dough structure formation and stability. The scald positively influenced the bread structure, producing uniform porosity and softness while reducing volume. The hardness of rye bread made with scald was 46.38% lower than that of bread without scald, corresponding to 2.30 and 4.29 N, respectively. Additional research is necessary, though, to optimize the ratio between scald and dough and achieve the desired bread quality. Barley scald did not provide a beneficial effect in reducing the hardness of barley bread since the hardness value was found to be 23.98% higher than that observed in bread without scald, corresponding to 19.97 and 22.28 N, respectively. Fermented rye scald can be considered a natural dough structure improver. For further research, rye scald could be examined as an enhancer for the structure and softness of barley dough. Overall, dough fermentation using sourdough and fermented rye scald is a suitable strategy, making it possible to use wholegrain flour to prepare 100% rye or barley entire-grain bread.

## Figures and Tables

**Table 1:** Chemical composition of rye and hull-less barley flour.

Item	Content (% dw)	
	Rye Flour	Hull-Less Barley Flour
Total carbohydrates	74.20	72.70
Starch	57.49	60.90
Crude fiber	1.80	0.75
Dietary fiber	15.80	12.00
Proteins	7.70	9.60
Ash	1.46	1.61

Note: % dw—% of dry weight.

**Table 2:** Ingredients used for the preparation of barley and rye dough, g.

Ingredient	RDNS	RDWS	BDNS	BDWS
Rye flour	1725	990	–	–
Barley flour	–	–	1725	990
FRS	–	2200	–	–
FBS	–	–	–	2200
Water	1465	–	1465	–
Sourdough D	149	149	149	149
Salt	17	17	17	17
Sugar	34	34	34	34
Total	3390	3390	3390	3390

Note: FRS—fermented rye scald; FBS—fermented barley scald; Sourdough D—rye sourdough for dough fermentation; RDNS—rye dough without fermented scald; RDWS—rye dough with fermented scald; BDNS—barley dough without fermented scald; BDWS—barley dough with fermented scald.

**Table 3:** Microbiological and physio-chemical characteristics of sourdough and scald.

Samples	LAB (log_10_ CFU g^−1^)	Yeasts (log_10_ CFU g^−1^)	pH	TTA (mL NaOH)
Sourdough S	7.1 ± 0.5 ^b^	2.6 ± 0.4 ^a^	3.68 ± 0.01 ^b^	14.5 ± 0.3 ^e^
Sourdough D	9.3 ± 0.7 ^c^	8.5 ± 0.2 ^b^	3.88 ± 0.03 ^c^	14.3 ± 0.1 ^f^
RS	4.9 ± 0.8 ^a^	2.5 ± 0.2 ^a^	6.92 ± 0.01 ^e^	1.4 ± 0.1 ^a^
FRS	7.4 ± 0.7 ^b^	2.9 ± 0.8 ^a^	3.61 ± 0.06 ^a^	12.5 ± 0.2 ^d^
BS	4.5 ± 0.5 ^a^	2.3 ± 0.1 ^a^	6.39 ± 0.02 ^d^	2.0 ± 0.1 ^b^
FBS	6.0 ± 0.6 ^ab^	2.5 ± 0.4 ^a^	3.67 ± 0.01 ^ab^	11.8 ± 0.1 ^c^

Note: Sourdough S—part of fermented scald received from the bakery for rye and barley scald; Sourdough D—rye sourdough for rye and barley dough; RS—rye scald after gelatinization and saccharification (2 h); FRS—fermented rye scald (24 h); BS—barley scald after gelatinization and saccharification (2 h); FBS—fermented barley scald (24 h); CFU—colony forming units; TTA—titratable acidity. Different letters (a–f) in the same column indicate significant differences (*p* ≤ 0.05) between the samples.

**Table 4:** Complex viscosity and loss factor at 1 rad s^−1^, 10 rad s^−1^, and 100 rad s^−1^ for rye and barley dough.

Dough	1 rad s^−1^		10 rad s^−1^		100 rad s^−1^	
	Complex Viscosity (Pa s)	Loss Factor (–)	Complex Viscosity (Pa s)	Loss Factor (–)	Complex Viscosity (Pa s)	Loss Factor (–)
RDNS	11,822 ± 3708 ^b^	0.52 ± 0.01 ^bc^	1489 ± 385 ^ab^	0.42 ± 0.01 ^c^	229 ± 54 ^c^	0.38 ± 0.01 ^ab^
BDNS	19,938 ± 12,734 ^c^	0.36 ± 0.01 ^ab^	1599 ± 329 ^ab^	0.32 ± 0.01 ^ab^	222 ± 42 ^c^	0.37 ± 0.01 ^ab^
RDWS	10,021 ± 2519 ^b^	0.45 ± 0.01 ^abc^	1228 ± 217 ^ab^	0.38 ± 0.01 ^bc^	186 ± 29 ^b^	0.38 ± 0.01 ^ab^
BDWS	16,500 ± 612 ^c^	0.31 ± 0.01 ^a^	1750 ± 25 ^b^	0.26 ± 0.01 ^a^	230 ± 1 ^c^	0.29 ± 0.01 ^a^
RDNS4H	9522 ± 6472 ^a^	0.69 ± 0.08 ^d^	766 ± 178 ^a^	0.56 ± 0.01 ^d^	128 ± 28 ^a^	0.52 ± 0.01 ^c^
BDNS4H	11,085 ± 826 ^b^	0.40 ± 0.03 ^ab^	1204 ± 113 ^ab^	0.34 ± 0.01 ^abc^	173 ± 12 ^b^	0.40 ± 0.02 ^b^
RDWS4H	6010 ± 850 ^a^	0.58 ± 0.01 ^cd^	831 ± 154 ^a^	0.42 ± 0.01 ^c^	132 ± 24 ^a^	0.43 ± 0.01 ^b^
BDWS4H	9347 ± 3283 ^a^	0.38 ± 0.07 ^ab^	1003 ± 171 ^ab^	0.31 ± 0.05 ^ab^	151 ± 43 ^a^	0.35 ± 0.06 ^ab^

Note: RDNS—rye dough without scalded rye flour; BDNS—barley dough without scalded barley flour RDWS—rye dough with scalded rye flour; BDWS—barley dough with scalded barley flour; RDNS4H—rye dough RDWS4H—rye dough with fermented rye scald after 4 h fermentation. Different letters (a–d) in the same column indicate significant differences (*p* ≤ 0.05) between the samples.

**Table 5:** Physico-chemical characteristics of wholegrain barley and rye bread without and with scald.

Parameter	RBNS	RBWS	BBNS	BBWS
pH	5.15 ± 0.06 ^d^	4.09 ± 0.02 ^a^	4.89 ± 0.02 ^c^	4.26 ± 0.01 ^b^
TTA, mL NaOH	5.0 ± 0.1 ^a^	10.4 ± 0.2 ^c^	5.3 ± 0.2 ^a^	7.8 ± 0.2 ^b^
a_w_	0.96 ± 0.01 ^b^	0.95 ± 0.01 ^a^	0.96 ± 0.01 ^b^	0.95 ± 0.01 ^a^
Moisture, %	48.04 ± 0.82 ^ab^	45.32 ± 1.93 ^a^	48.28 ± 0.23 ^b^	45.99 ± 0.75 ^ab^
Porosity, %	67.0 ± 1.1 ^d^	58.8 ± 2.0 ^c^	48.4 ± 1.3 ^b^	39.9 ± 1.2 ^a^
Specific volume, cm^3^ g^−1^	1.85 ± 0.02 ^d^	1.61 ± 0.01 ^c^	1.28 ± 0.02 ^b^	1.13 ± 0.05 ^a^
Hardness, N	4.29 ± 1.13 ^a^	2.30 ± 0.55 ^a^	17.97 ± 2.49 ^b^	22.28 ± 3.18 ^c^

Note: TTA—titratable acidity, mL of 0.1 N NaOH; a_w_—water activity; RBNS—rye bread without scald; RBWS—rye bread with scald; BBNS—barley bread without scald; BBWS—barley bread with scald. Different letters (a–d) in the same row indicate significant differences (*p* ≤ 0.05) between the samples.

## Data Availability

Data is contained within the article.
